# Natural History of Postoperative Adding-On in Adolescent Idiopathic Scoliosis: What Are the Risk Factors for Progressive Adding-On?

**DOI:** 10.1155/2018/3247010

**Published:** 2018-03-29

**Authors:** Xiaodong Qin, Chao Xia, Leilei Xu, Fei Sheng, Huang Yan, Yong Qiu, Zezhang Zhu

**Affiliations:** Department of Spine Surgery, The Affiliated Drum Tower Hospital of Nanjing University Medical School, Nanjing, China

## Abstract

**Purpose:**

To investigate the natural history of distal adding-on in adolescent idiopathic scoliosis (AIS) and to identify risk factors for its progression.

**Methods:**

Sixty-one AIS patients with distal adding-on occurrence were included. We further classify distal adding-on into progressive and nonprogressive group according to its natural evolution. The first radiograph indicating initiation of adding-on (primary adding-on) and the last follow-up radiograph were compared in terms of the deviation of the first vertebra below instrumentation from the CSVL and the angulation of the first disc below instrumentation. Compared to primary adding-on, progressive adding-on was defined as a further increase of deviation > 5 mm or a further increase of angulation > 5°. Risk factors associated with the progression of adding-on were analyzed.

**Results:**

Among 61 patients diagnosed with distal adding-on, 24 (39.3%) were progressive and 37 (60.7%) were nonprogressive. Lower Risser grade, open triradiate cartilage, and lowest instrumented vertebra (LIV) proximal to Substantially Stable Vertebra (SSV) were found to be significantly associated with the progressive adding-on. Besides, the distal adding-on was more likely to progress for patients with higher left shoulders than right ones after surgery.

**Conclusions:**

The risk factors for the progression of adding-on included skeletal immaturity, LIV proximal to SSV, and higher left shoulders after surgery.

## 1. Introduction

Adolescent idiopathic scoliosis (AIS) is a 3-dimensional deformity of the spine that constitutes the most common type of spinal deformity around puberty. Surgical treatment is usually performed in patients with the curve exceeding 45° to prevent further curve progression and obtain a balanced spine [1]. For some of AIS patients with Lenke 1A and 2A curves, only main thoracic curve needs to be corrected and fused since the flexible lumbar curve can provide postoperative compensation [2–5]. However, some patients may have postoperative coronal decompensation although lumbar curves can improve spontaneously after selective thoracic fusion. Of particular concern is the “adding-on” phenomenon caudal to the fusion, defined as an increase in the number of vertebrae within the distal curve from the first erect radiograph postoperatively to the last follow-up [6]. Distal adding-on could lead to increased coronal decompensation and disc wedging, which in turn could result in degenerative changes later in life and further surgical intervention [7].

Risk factors associated with adding-on have been well documented in previous studies, such as lowest instrumented vertebra (LIV) selection, apical translation, and skeletal maturity [6–10]. Among them, the inappropriate selection of LIV seems to be the major cause [6, 8–10]. In previous literature, nearly 6-7% of AIS patients with adding-on need revision surgery for progressive adding-on [10, 11]. However, few studies have specifically focused on the progression of distal adding-on, which is often accompanied by unsatisfactory clinical outcome and high risk of revision surgery in clinical practice. The purpose of this study is to investigate the natural history of postoperative distal adding-on in AIS patients undergoing selective thoracic fusion and to identify the risk factors related to the progression of this complication.

## 2. Materials and Methods

### 2.1. Patients Inclusion

**Figure 1:**
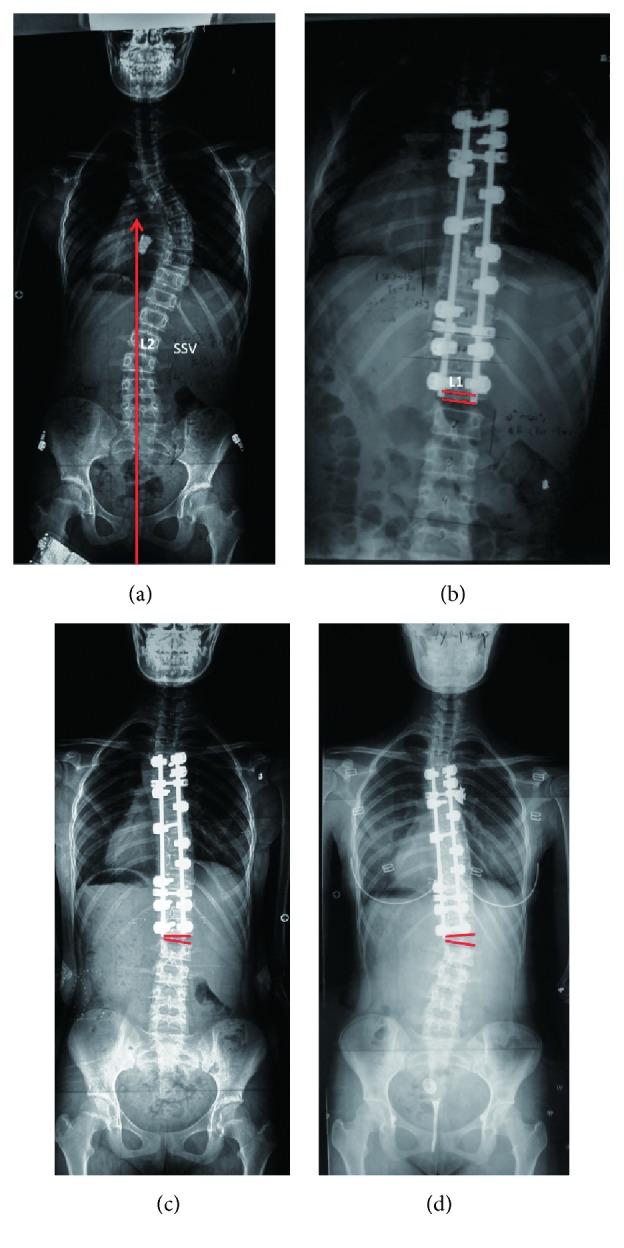
(a) A 13-year-old girl with Lenke 1A curve. L2 was SSV; Risser grade was 0. (b) First erect radiograph postoperatively with fusion to L1, one level proximal to SSV. (c) 3-month postoperative radiograph shows adding-on; the angulation of the first disc below the instrumentation was 6.8°. (d) 4-year postoperative radiograph shows adding-on progressed; the disc angulation increased to 12.2°.

**Figure 2:**
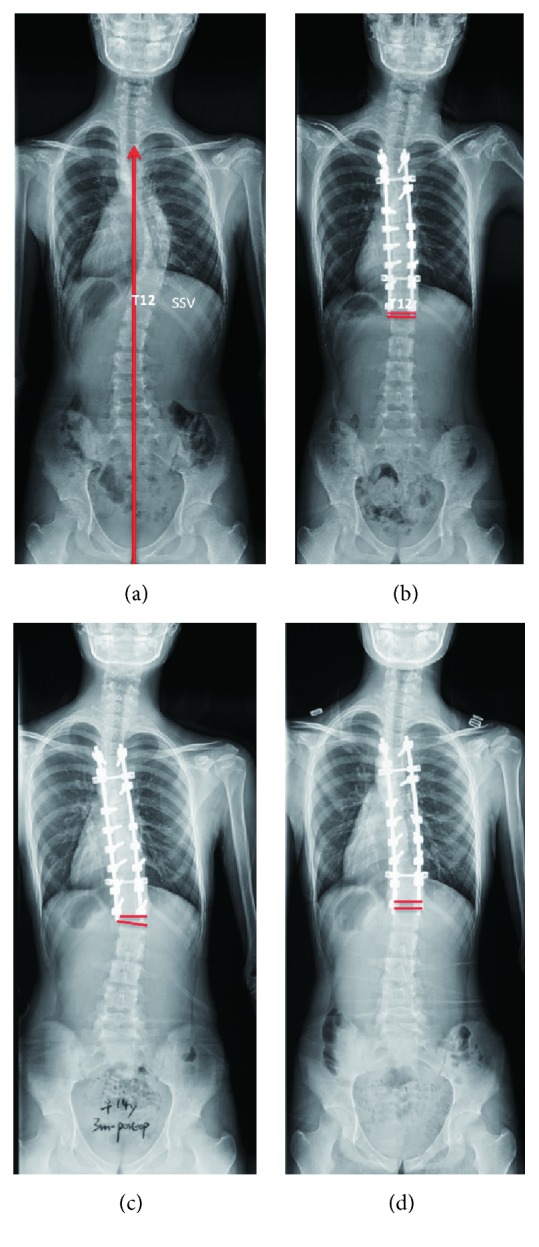
(a) A 14-year-old girl with Lenke 1A curve. T12 was SSV; Risser grade was 3. (b) First erect radiograph postoperatively with fusion to T12. (c) 3-month postoperative radiograph shows adding-on; the angulation of the first disc below the instrumentation was 10.6°. (d) 2.4-year postoperative radiograph shows adding-on decreased; the disc angulation decreased to 2.8°.

This study was approved by the University Institutional Review Board. A cohort of 284 patients who received selective posterior thoracic fusion surgery from 2006 to 2015 were retrospectively reviewed. Patients were recruited into this study with the following criteria: (1) age from 11 to 18 years; (2) Lenke type 1A or 2A; (3) distal adding-on occurring during the follow-up; (4) follow-up more than 2 years. Primary distal adding-on was defined as an increase in the number of vertebrae included within the distal curve from the first erect radiograph to the follow-up radiograph, with (1) an increase of more than 5 mm in the deviation of the first vertebra below the instrumentation from the CSVL or (2) an increase of more than 5° in the angulation of the first disc below the instrumentation [6]. Among the 284 patients reviewed, 61 (21.5%) with primary distal adding-on occurrence during follow-up were included in the study. These patients were further classified into progressive and nonprogressive group according to the natural evolution of adding—during the follow-up. The first radiograph indicating the initiation of adding-on (primary adding-on) and the last follow-up radiograph were compared in terms of the deviation of the first vertebra below the instrumentation from the CSVL and the angulation of the first disc below the instrumentation. Compared to the primary adding-on, the progressive adding-on was defined as a further increase of deviation > 5 mm or a further increase of angulation > 5° (Figure 1). Nonprogressive adding-on was defined as the increase of deviation ⩽ 5 mm and the increase of angulation ⩽ 5° (Figure 2).

### 2.2. Clinical Features

The baseline characteristics were recorded including age, sex, Risser grade, triradiate cartilage, curve magnitude, and the distance between LIV and Substantially Stable Vertebra (SSV). As reported in the previous study [12], SSV was defined as the Substantially Touched Vertebra (STV) or one level distal to non-Substantially Touched Vertebra (nSTV). STV was defined as the last touching vertebra (LTV) where center sacral vertical line (CSVL) either was between the pedicles or intersected the pedicle; nSTV was defined as the LTV where CSVL touched the corner of the vertebra lateral to the pedicle border [12]. The distance between LIV and SSV was defined as the number of vertebrae between LIV and SSV.

### 2.3. Radiographic Measurements

Radiographic measurements were performed on preoperative upright posteroanterior (PA) and lateral radiographs as well as right and left supine side-bending coronal radiographs. In addition, standing PA and lateral radiographs obtained immediately after surgery and at every 3-month visit were also evaluated. The measured radiographical parameters included the Cobb angles of the proximal thoracic curves (PT), main thoracic curves (MT), and lumbar curves, the apical vertebral translation of the MT curve, coronal balance, sagittal balance, and trunk shift. The clavicle angle (CA), radiographical shoulder height (RSH), and T1 tilt angle were also evaluated [8]. Coronal balance was determined according to the distance between the coronal C7 plumbline (C7PL) and CSVL, with a value > 20 mm defined as imbalance [13]. Sagittal balance was determined according to the distance between C7PL and the posterior sacral vertical line (PSVL), with a value > 50 mm defined as imbalance [14]. Trunk shift was measured between CSVL and vertical trunk reference line (VTRL) [15]. Curve flexibility was calculated by the following equation: (preoperative Cobb angle − side-bending Cobb angle)/preoperative Cobb angle × 100 (%) [15]. Curve correction was calculated as follows: (preoperative Cobb angle − postoperative Cobb angle)/preoperative Cobb angle × 100 (%) [16]. RSH = left shoulder height – right shoulder height. Patients were classified into three groups according to RSH: (1) L > R group: RSH > 10 mm; (2) L = R group: −10 mm ≤ RSH ≤ 10 mm; (3) L < R group: RSH ≤ −10 mm. All measurements were performed by Surgimap (Spine Software, version 2.1.2, New York, NY, USA). Two of the authors completed the measurement together. In addition, 20 patients were randomly selected to determine the intra- and interobserver variability of the measurement. All the radiographic parameters of the selected patients were measured by the authors and then repeated twice. There were strong intraobserver and interobserver agreements for all the parameters with the kappa correlation coefficients exceeding 0.8. Therefore, the measured data were reliable, and the mean values of the data measured by the two investigators were recorded.

*Evaluation of Quality of Life.* Patients were all required to complete the Scoliosis Research Society (SRS-22) questionnaires at the last follow-up. The SRS-22 covers five domains including function/activity, pain, self-perceived image, satisfaction with treatment, and mental health. Questions of each domain have 5 verbal response alternatives ranging from 1 to 5, with a value of 5 indicating the best outcome. Results of SRS-22 questionnaires are expressed using the mean value for each domain, as calculated by dividing total sum of the domain with the number of items answered [17].

*Statistical Analyses.* The Student *t*-test, chi-square test, or Fisher's exact test was used to compare continuous or categorical variables between patients with progressive and nonprogressive distal adding-on. Statistical analyses were performed with SPSS 20.0 statistical software (SPSS Inc., Chicago, IL). A *P* value less than 0.05 was considered statistically significant.

## 3. Results

### 3.1. Clinical and Radiographic Features of the Patients

**Figure 3:**
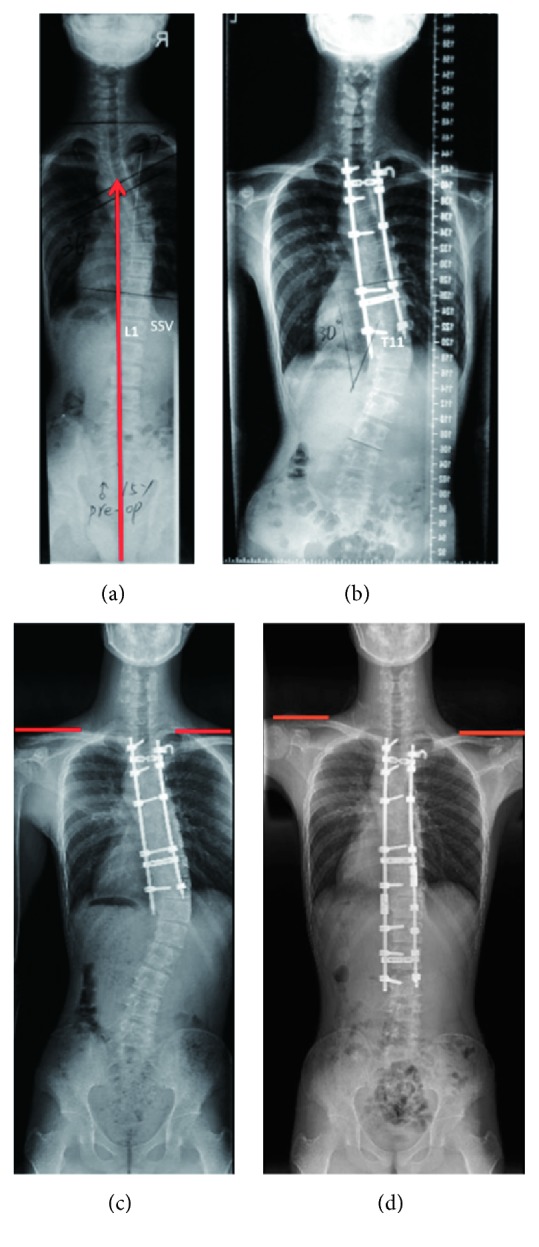
(a) A 15-year-old boy with Lenke 2A curve, who received primary surgery in another hospital. L1 was SSV; Risser grade was 2. (b) 2-year postoperative radiograph shows adding-on; the deviation of the first vertebra below the instrumentation (LIV + 1) from the CSVL was 45.5 mm. LIV was 2 levels proximal to SSV. (c) 4-year postoperative radiograph shows adding-on progressed; the LIV + 1 deviation increased to 53.2 mm. Shoulders were level with RSH of 4.24 mm. (d) Postrevision radiograph shows left shoulder elevated with RSH of 37.44 mm.

Most of the primary distal adding-on occurred within 3 months after surgery (85.2%). Among 61 patients diagnosed with distal adding-on, 41 had Lenke 1A curves and 20 had Lenke 2A curves. According to the natural evolution of adding-on, 24 (39.3%) patients were included in the progressive group and 37 (60.7%) patients were included in the nonprogressive group. The mean age at the time of surgery was 15.1 ± 2.1 years. The mean follow-up time was 42.1 ± 17.6 months (24–96 months). The mean Risser grade was 2.8 ± 1.2. Twenty-six patients had open triradiate cartilage (OTRC) and 35 patients had closed triradiate cartilage (CTRC). The mean preoperative Cobb angles of the proximal thoracic, thoracic, and lumbar curves were 28.3 ± 5.5°, 50.1 ± 9.9°, and 23.1 ± 6.2°, with a mean flexibility of 27.5 ± 13.7%, 56.2 ± 17.7%, and 83.5 ± 17.1%, respectively. At the last follow-up, they were corrected to 17.8 ± 8.1°, 14.9 ± 8.8°, and 9.0 ± 4.9°, with a mean correction rate of 35.1 ± 24.2%, 69.9 ± 14.9%, and 59.4 ± 37.8%, respectively. There were 8 cases of coronal imbalance and 4 cases of sagittal imbalance, respectively. One patient required revision surgery for severe progressive adding-on (Figure 3).

### 3.2. Risk Factors for Progressive Distal Adding-On

As shown in Table 1, patients in the two groups were matched in terms of preoperative clinical and radiographical factors except for the Risser grade, triradiate cartilage, and the level of LIV. The mean Risser grade was 1.88 ± 1.45 in the progressive group and 3.48 ± 1.14 in the nonprogressive group. Lower Risser grade was found to be significantly associated with the progressive adding-on (*P* < 0.001). 62.5% patients had OTRC in the progressive group, while 29.7% patients had OTRC in the nonprogressive group (*P* = 0.011). 50.0% patients had the LIV proximal to SSV in the progressive group, while 8.1% patients had the LIV proximal to SSV in the nonprogressive group (*P* < 0.001). Moreover, as shown in Table 2, concerning the immediately postoperative RSH, 37.5% patients had higher left shoulder (L > R) in the progressive group, while 8.1% patients had higher left shoulder (L > R) in the nonprogressive group (*P* = 0.008). Besides, 8.3% patients had higher right shoulder (L < R) in the progressive group, while 40.5% patients had higher right shoulder (L < R) in the nonprogressive group (*P* = 0.008). There were no significant differences between the two groups as for other immediately postoperative radiographical parameters.

### 3.3. Comparison of SRS Scores between Progressive and Nonprogressive Groups

The mean pain score of SRS-22 questionnaire at the last follow-up was significantly lower in the progressive group than that in the nonprogressive group (4.2 ± 0.5 versus 4.5 ± 0.5, *P* = 0.013), indicating that the painful conditions of the patients with progressive adding-on gradually worsened during follow-up. As for other domains including self-image, general function, mental health, and satisfaction, there were no significant differences between the two groups (Table 3).

## 4. Discussion

Distal adding-on is a common complication in AIS patients with a prevalence ranging from 12.9% to 51.1% [6, 8–11, 18, 19]. Adding-on may have adverse effects on the lumbar spine in the long term or even require revision surgery. Cao et al. [10] analyzed 116 Lenke 2A patients, and postoperative distal adding-on was observed in 16 patients. 1 patient required revision surgery for severe adding-on with progressive thoracolumbar curve to the right and for low back pain. Yang et al. [11] reviewed 98 patients with Lenke 1A and 2A curve, and postoperative distal adding-on was observed in 16.3% of them. Great progression of adding-on was observed in 2 patients (12.5%) in adding-on group, among whom 1 patient received the revision surgery.

In this study, for the first time, the phenomenon of adding-on was classified into progressive adding-on and nonprogressive adding-on according to its natural evolution. Progressive adding-on was determined through comparison between the first radiograph indicating the initiation of adding-on (primary adding-on) and the last follow-up radiograph, as either a further increase > 5 mm in the deviation of the first vertebra below the instrumentation from CSVL or a further increase > 5° in the angulation of the first disc below the instrumentation. In our case series, distal adding-on was observed in 61 out of 284 (21.5%) patients at the final follow-up, among whom 24 (39.3%) patients were included in the progressive group and 37 (60.7%) patients were included in the nonprogressive group.

Since the progression of adding-on might be associated with the unsatisfactory clinical outcomes, it is of great importance to identify the related risk factors. In the current study, we found that the selection of LIV was significantly associated with the progression of distal adding-on. When LIV was cranial to SSV, the adding-on is more likely to progress, indicating that a short fusion level might be a risk factor for progression. We hypothesized that the lumbar curve would partially correct shortly after selective thoracic fusion. During the follow-up, the lumbar curve could gradually move back to its original location if the instrumentation was too short to maintain the spontaneous lumbar correction. Consequently, the distal adding-on occurred and progressed until the lumbar curve returned to original position. Besides, several authors have found that selection of LIV was highly associated with the onset of distal adding-on. Matsumoto et al. [8] found the LIV proximal to the LTV was significantly associated with adding-on and recommended extending the LIV at least to the LTV to avoid postoperative adding-on. Our previous study found the distance between LIV and STV/nSTV + 1 was a significant factor associated with postoperative distal adding-on. Selecting STV or nSTV + 1 as LIV could yield a promising outcome for Lenke 1A scoliosis patients [12]. Since a short fusion segment is correlated with the onset and progression of postoperative distal adding-on, the LIV should be selected distally. However, it is important to conserve lumbar mobility and growth potential, so the LIV should not be selected distally without limitation. Therefore, the optimal selection of LIV should take into consideration the incidence of adding-on and conservation of the lumbar mobility.

Besides, Risser sign and OTRC were found to be significantly associated with the progression of adding-on in our study, indicating that skeletal immaturity may also be a risk factor for progression. For skeletal immature patients, surgical treatment may achieve greater correction; however, in some cases it may cause biomechanical changes leading to a secondary progression of distal adding-on. In previous literature, skeletal maturity was also found to be associated with distal adding-on. Sponseller et al. [20] found AIS patients with OTRC had greater loss of the spontaneous lumbar curve correction after selective posterior spinal fusion. Schlechter et al. [21] reported that less mature patients were more likely to experience the distal adding-on. Herein, during the strategy planning for the selection of LIV in skeletal immature AIS patients, an extra distal level may be considered to reduce the risk of progressive adding-on.

**Figure 4:**
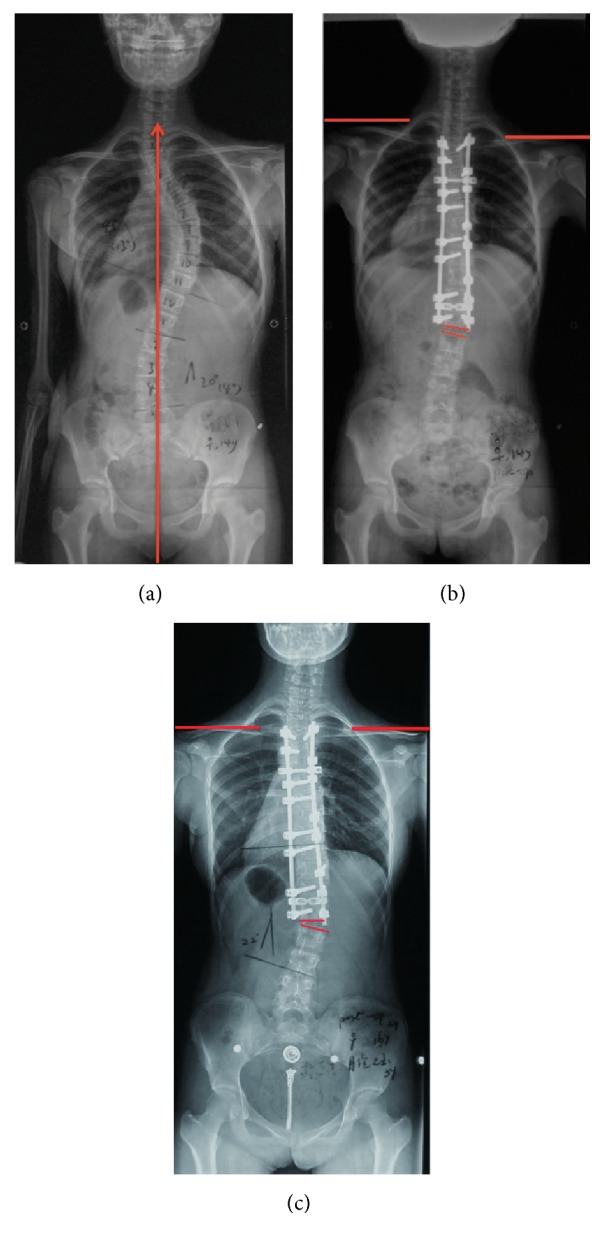
(a) Preoperative radiograph of a 14-year-old boy with Lenke 1A curve. (b) 3-month postoperative radiograph shows adding-on with the disc angulation of 6.1°. Left shoulder elevated with RSH of 21.5 mm. (c) 2-year postoperative radiograph shows adding-on progressed with the disc angulation of 12.1°. Shoulders were level with RSH of 5.5 mm.

Previous literature reported that the postoperative shoulder balance and postoperative distal adding-on were significantly associated with each other. Since the thoracic spine was fixed after surgery, the lumbar spine would compensate for postoperative shoulder imbalance (PSI) [22]. However, the mechanism underlying the relationship between distal adding-on and PSI remains unknown. In this study, we found distal adding-on might progress during follow-up if left shoulder was higher than right side after surgery, while it might be stable or improved if the right shoulder was higher than left one. There was an interesting phenomenon: When distal adding-on progressed, the right shoulder might elevate. Hence, patients with higher left shoulders after surgery could rebalance the shoulders by progressive distal adding-on (Figure 4). On the contrary, when distal adding-on improved, the left shoulder might elevate (Figure 3). Therefore, for some adding-on patients with higher right shoulders after surgery, the distal adding-on might keep stable or improve during follow-up.

The relationship between distal adding-on and the Quality of Life remained unknown. Matsumoto et al. [8] found there was no significant difference in postoperative SRS scores between patients with and without adding-on. While Upasani et al. [23] concluded that the SRS-22 scores of the appearance domain at two years after surgery were significantly worse in the deformity progression group. In our study, AIS patients with progressive adding-on seemed to have worse pain scores. Besides, one patient required revision surgery for severe progression of adding-on. Hence, the progression of distal adding-on might exert adverse effects on the painful conditions and increase the reoperation rate.

Two limitations of our study should be addressed. First, our findings could be biased because of the relatively small number of patients with adding-on. Future study with a larger sample size is warranted for a sound conclusion. Second, SRS-22 scores before surgery were not evaluated in this study, which made comparison between preoperative and postoperative scores impossible.

## 5. Conclusion

The distal adding-on could be classified into progressive adding-on and nonprogressive adding-on according to its natural evolution. Among patients diagnosed with distal adding-on, the incidence of progressive and nonprogressive adding-on was 39.3% and 60.7%, respectively. Skeletally immature patients with short fusion level and higher left shoulders after surgery seem to be more likely to have progressive adding-on, which exerts adverse effects on the pain scores of SRS-22.

## Figures and Tables

**Table 1:** Clinical and preoperative radiographical data.

	Progressive (*n* = 24)	Nonprogressive (*n* = 37)	*P*
Clinical data			
Sex (female/male)	19/5	32/5	0.451
Age (year)	14.62 ± 2.15	15.33 ± 2.11	0.103
Follow-up (month)	43.81 ± 20.62	40.98 ± 15.91	0.274
Risser	1.88 ± 1.45	3.48 ± 1.14	<0.001
Triradiate cartilage (OTRC/CTRC)	15/9	11/26	0.011
LIV-SSV			
<0	12	3	<0.001
0	9	12	
>0	3	22	
Preoperative radiographical data			
PT curve (°)	28.99 ± 4.91	27.84 ± 6.03	0.219
Flexibility (%)	28.42 ± 13.21	26.92 ± 14.32	0.341
MT curve (°)	51.12 ± 10.54	49.52 ± 9.43	0.269
Flexibility (%)	53.24 ± 18.18	58.12 ± 17.12	0.146
Lumbar curve (°)	22.37 ± 7.21	23.51 ± 5.66	0.247
Flexibility (%)	82.93 ± 15.89	83.91 ± 18.12	0.415
Thoracic kyphosis (°)	11.52 ± 6.12	13.98 ± 8.21	0.107
Lumbar lordosis (°)	48.59 ± 6.35	47.58 ± 7.91	0.301

OTRC: open triradiate cartilage; CTRC: closed triradiate cartilage; LIV: lower instrumented vertebra; SSV: Substantially Stable Vertebra; LIV-SSV < 0: LIV proximal to SSV; LIV-SSV = 0: LIV at SSV; LIV-SSV > 0: LIV distal to SSV.

**Table 2:** Postoperative radiographical data.

	Progressive (*n* = 24)	Nonprogressive (*n* = 37)	*P*
PT curve (°)	16.15 ± 5.82	15.03 ± 6.35	0.245
Correction rate (%)	44.19 ± 21.12	45.91 ± 19.27	0.372
MT curve (°)	12.94 ± 6.75	11.96 ± 5.31	0.265
Correction rate (%)	74.38 ± 12.02	75.79 ± 8.98	0.301
Lumbar curve (°)	8.78 ± 4.89	8.54 ± 3.91	0.416
Correction rate (%)	60.83 ± 21.59	63.53 ± 18.12	0.301
Thoracic kyphosis (°)	17.36 ± 6.04	16.99 ± 7.24	0.418
Lumbar lordosis (°)	48.47 ± 9.74	46.93 ± 9.18	0.267
Coronal balance (mm)	12.94 ± 11.39	14.13 ± 10.04	0.335
Sagittal balance (mm)	27.95 ± 16.93	26.13 ± 18.32	0.349
Apical translation (mm)	15.04 ± 8.45	17.94 ± 11.32	0.143
Clavicle angle (°)	1.89 ± 1.21	2.02 ± 1.32	0.349
T1 tilt angle (°)	5.57 ± 3.12	5.84 ± 3.83	0.387
Trunk shift (mm)	12.32 ± 8.13	13.03 ± 9.14	0.379
RSH			
L > R	9	3	0.003
L = R	13	19	
L < R	2	15	

All postoperative parameters were measured immediately after operation. RSH = left shoulder height – right shoulder height; L > R defined as left shoulder height – right shoulder height > 10 mm; L = R defined as 10 mm ≥ left shoulder height – right shoulder height ≥ −10 mm; L < R defined as left shoulder height – right shoulder height ≤ −10 mm.

**Table 3:** Adding-on and SRS-22 scores.

	Progressive (*n* = 24)	Nonprogressive (*n* = 37)	*P*
Function	4.4 ± 0.7	4.5 ± 0.6	0.297
Pain	4.2 ± 0.5	4.5 ± 0.5	0.013
Self-image	4.1 ± 0.4	4.2 ± 0.5	0.231
Mental health	4.2 ± 0.7	4.2 ± 0.5	0.474
Satisfaction	4.1 ± 0.5	4.2 ± 0.7	0.273
